# Analysis of REST binding sites with canonical and non-canonical motifs in human cell lines

**DOI:** 10.1186/s12920-024-01860-4

**Published:** 2024-04-17

**Authors:** Jaejoon Choi, Eunjung Alice Lee

**Affiliations:** 1 Division of Genetics and Genomics, Boston Children’s Hospital and Harvard Medical School, Boston, MA USA; 2 Manton Center for Orphan Disease Research, Boston Children’s Hospital, Boston, MA USA; 3 The Broad Institute of Harvard and MIT, Cambridge, MA USA

## Abstract

**Background:**

Repressor element 1 (RE1) silencing transcription factor (REST) is a transcriptional repressor abundantly expressed in aging human brains. It is known to regulate genes associated with oxidative stress, inflammation, and neurological disorders by binding to a canonical form of sequence motif and its non-canonical variations. Although analysis of genomic sequence motifs is crucial to understand transcriptional regulation by transcription factors (TFs), a comprehensive characterization of various forms of RE1 motifs in human cell lines has not been performed.

**Results:**

Here, we analyzed 23 ENCODE REST ChIP-seq datasets from diverse human cell lines and identified a non-redundant set of 68,975 loci with ChIP-seq peaks. Our systematic characterization of these binding sites revealed that the canonical form of REST binding motif was found primarily in ChIP-seq peaks shared across multiple cell lines, while non-canonical forms of motifs were identified in both cell-line-specific binding sites and those shared across cell lines. Remarkably, we observed a notable prevalence of non-canonical motifs that corresponded to half segments of the canonical motif. Furthermore, our analysis unveiled the presence of cell-line-specific REST binding patterns, as evidenced by the clustering of ChIP-seq experiments according to their respective cell lines. This observation underscores the cell-line specificity of REST binding at certain genomic loci, implying intricate cell-line-specific regulatory mechanisms.

**Conclusions:**

Overall, our study provides a comprehensive characterization of REST binding motifs in human cell lines and genome-wide RE1 motif profiles. These findings contribute to a deeper understanding of REST-mediated transcriptional regulation and highlight the importance of considering cell-line-specific effects in future investigations.

**Supplementary Information:**

The online version contains supplementary material available at 10.1186/s12920-024-01860-4.

## Background

Repressor element 1 (RE1) silencing transcription factor (REST), also known as Neural Restrictive Silencing Factor (NRSF) is an essential transcriptional repressor gene [1]. REST has been found to be highly expressed in aging human brains and regulates genes that are involved in oxidative stress, inflammation, and neurological disorders [2]. REST has a zinc finger domain that binds to 21 bp RE1 nucleotides and the composition of this RE1 motif has been studied extensively [3–7]. The canonical RE1 motif contains a 2-bp non-conserved residue between two end segments. However, non-canonical RE1 motifs have variations in the length of the middle insertion between the two segments [8, 9], orientation or composition of the two segments [6], and presence of just one versus both segments [6, 10]. Rockowitz et al. [11] compared REST binding sites of 15 different human cell lines and McGann et al. [12] analyzed REST binding sites on three different human brain tissues; however, these studies analysed only canonical or limited types of non-canonical RE1 motifs.

In our study, we performed a systematic analysis of REST binding sites using ChIP-seq data from various human cell lines. Our comprehensive analysis of ENCODE [13, 14] ChIP-seq data for 23 human cell lines identified genome-wide RE1 motif profiles as well as the characteristics of the REST binding sites.

## Results

### Identification of REST binding sites

We downloaded 23 REST ChIP-seq datasets of various human cell lines from the ENCODE database [13, 14] for genome-wide analysis of REST binding sites. ChIP-seq peaks were merged, and peaks in ENCODE blacklist regions [15] or High Occupancy Target (HOT) regions [16] were filtered out, since those regions are considered to be artifacts [15, 16]. Among 73,326 merged ChIP-seq peaks, 4,351 peaks overlapping into these regions were filtered out, and 68,975 peaks remained after the filtration.

**Fig. 1:**
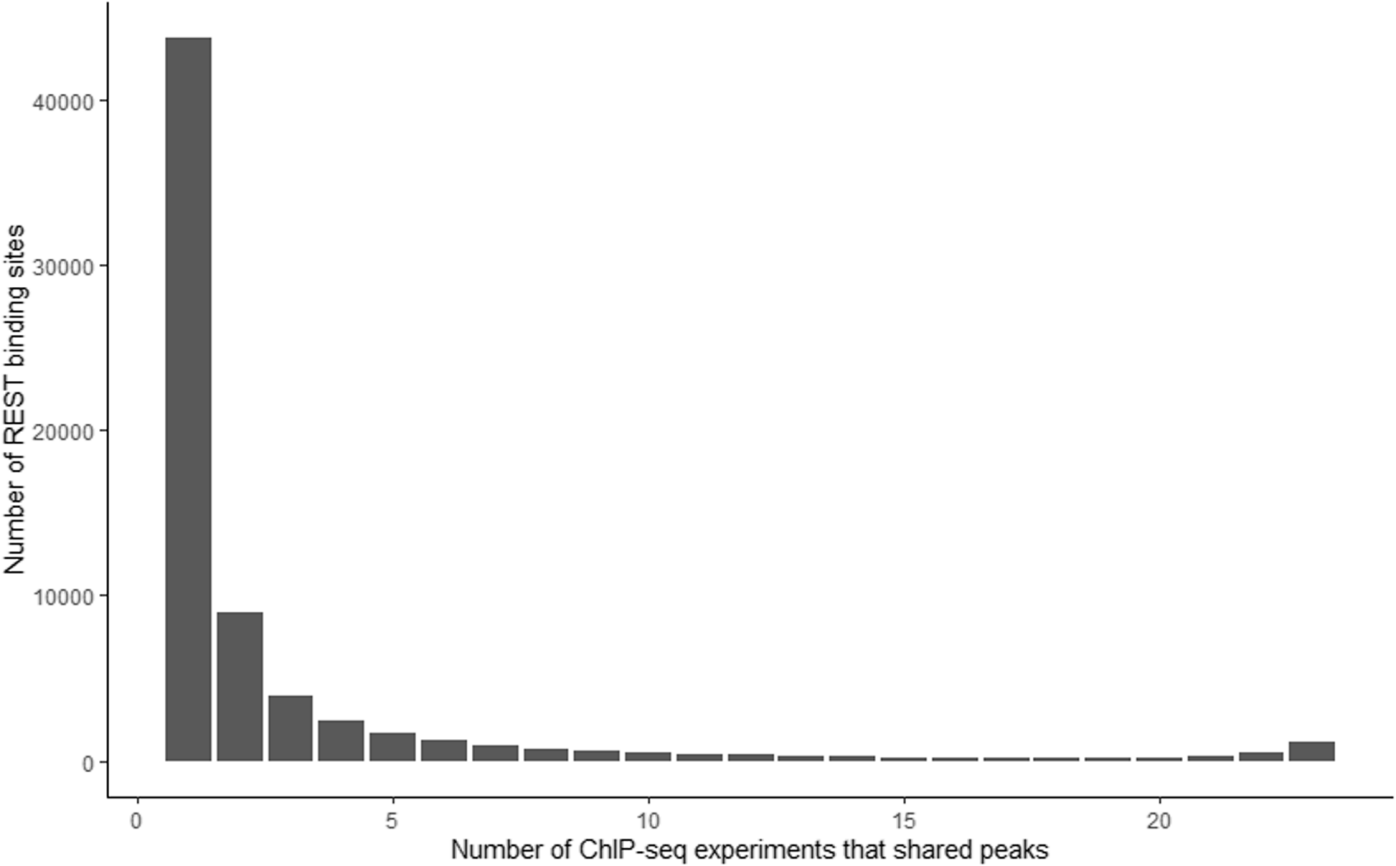
REST ChIP-seq peaks. Bar plots depict the number of REST binding sites according to the number of ChIP-seq experiments showing the binding peaks for a total of 68,975 binding sites from 23 ENCODE human REST ChIP-seq experiments across multiple cell lines

The number of peaks decreased until the number of ChIP-seq experiments that shared peaks reached 19 (Fig. 1). Only 2.8% of all peaks (1,920 out of 68,975) appeared in more than 90% of the ChIP-seq experiments (21 out of 23). Some of these peaks that were shared in a few ChIP-seq experiments might be REST binding sites that have cell-line specific binding affinity, but many peaks unique to single experiments might be experimental artifacts [17]. 63.4% (43,738 of 68,975) of the identified peaks were uniquely found in single experiments, and these singleton peaks were excluded in downstream analyses.

### Annotation of canonical and non-canonical RE1 motifs

**Fig. 2:**
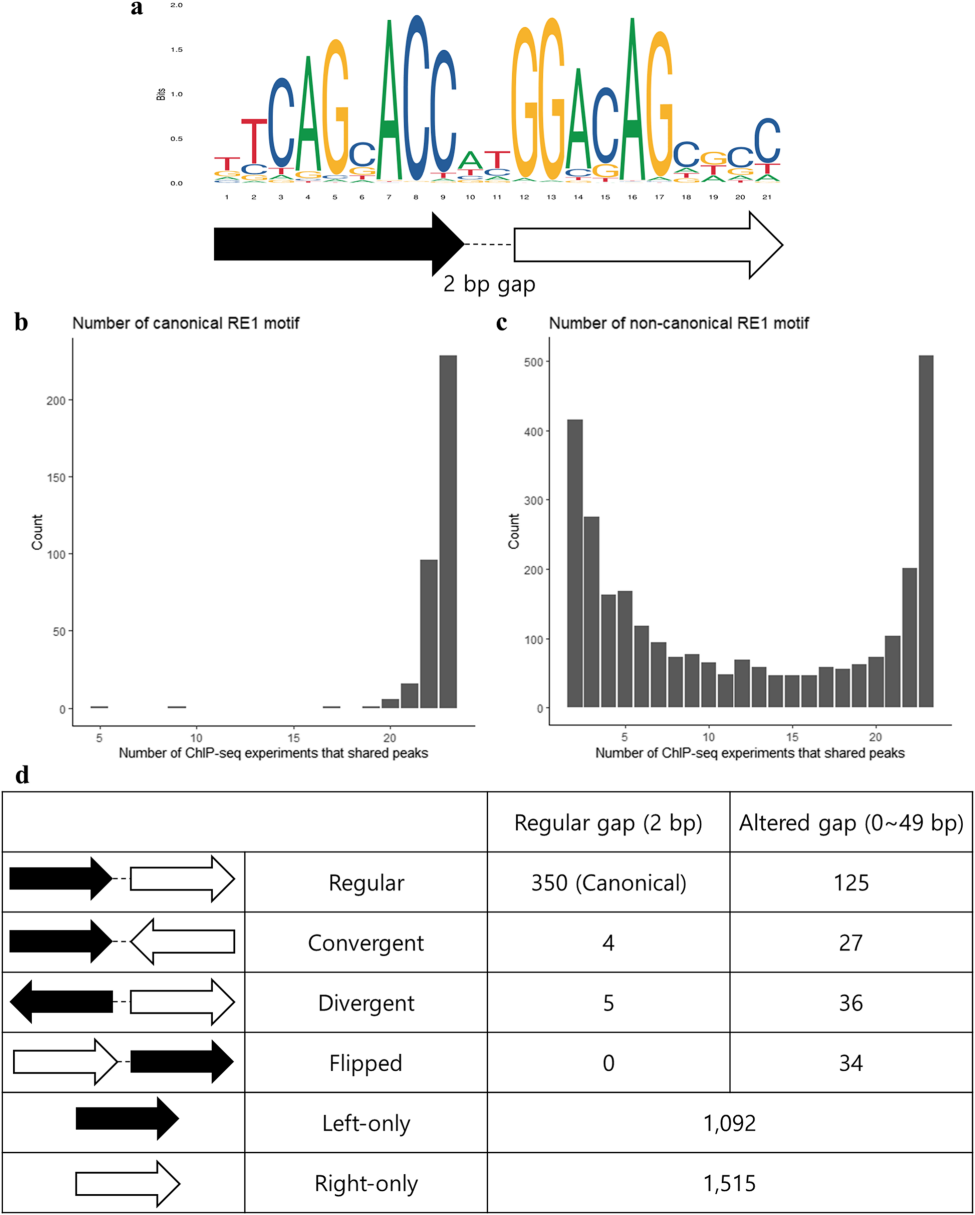
Canonical and non-canonical forms of RE1 motifs.  **a** Consensus RE1 motif. The arrows at the bottom indicate two segments of the RE1 motif. **b** The numbers of REST binding sites with the canonical RE1 motif by the numbers of ChIP-seq experiments showing the binding sites are shown as bar plots. **c** The number of REST binding sites with non-canonical RE1 motifs by their numbers of shared ChIP-seq experiments are shown as bar plots. **d** Both canonical and non-canonical RE1 motifs with different orientation, composition and gap length (‘Altered gap’ does not include 2 bp gap) are shown with their numbers of occurrence in ENCODE REST ChIP-seq experiments

The zinc finger domain of REST binds to the RE1 sequence motif. The canonical form of the RE1 motif is 21-bp long, which is divided into two conserved segments with a 2-bp gap between them (Fig. 2a). Non-canonical forms of the RE1 motif are composed of those two segments with different length of gaps between the two segments, different orientation of one segment (‘Convergent’ or ‘Divergent’), different order of segments (‘Flipped’), or even loss of one segment (‘Left-only’ or ‘Right-only’) [6].

**Fig. 3:**
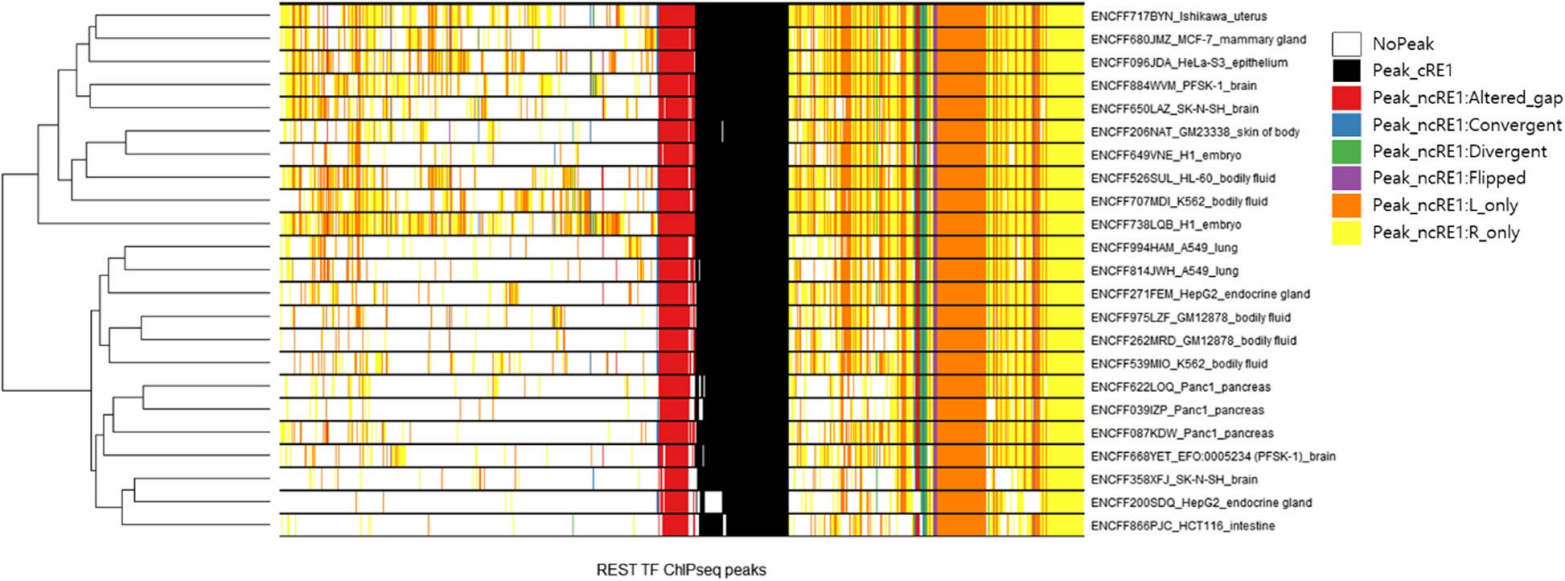
Recurrence of REST binding loci with canonical and non-canonical RE1 motifs across ENCODE experiments. Among 68,975 REST ChIP-seq peaks from 23 different ENCODE REST ChIP-seq experiments, 4,072 peaks that have RE1 motifs were selected. The presented heatmap shows genome-wide RE1 motif profiles for these 4,072 selected RE1 motif sites. Each row corresponds to a specific experiment, whereas each column represents a distinct ChIP-seq peak. The axes are clustered and ordered based on the clustered outcomes. The ChIP-seq experiments are identified through a three-segmented nomenclature, comprising the ENCODE identifier, cell-line name, and tissue name. Color key of heatmap − 1) White: ‘NoPeak’ – no ChIPseq peak was found in the relevant genomic region, 2) Black: ‘Peak_cRE1’ – ChIPseq peak was found in the relevant genomic region with canonical RE1 motif, and 3) Other colors: ‘Peak_ncRE1’ – ChIPseq peak was found in the relevant genomic region with non-canonical RE1 motifs; Red (Altered_gap), Blue (Convergent), Green (Divergent), Purple (Flipped), Orange (L_only), and Yellow (R_only)

Out of 25, 237 REST binding sites excluding singleton peaks, we identified 350 sites with canonical RE1 motifs (Fig. 2b and Supplementary Table 2). Among them, 347 (99%) binding sites appeared in 19 out of 23 (83%) ChIP-seq experiments (Fig. 2b). This is consistent with previous reports that canonical/consensus RE1 motifs appear in commonly found REST ChIP-seq peaks, and not in tissue-specific peaks [12]. We also identified various forms of non-canonical RE1 motifs from REST binding sites (Fig. 2c-d and Supplementary Table 3). Unlike canonical forms, non-canonical motifs appeared in both cell-line specific (i.e., those detected in a small number of ChIP-seq experiments) sites and universal sites (Fig. 3). For RE1 half motifs (‘Left-only’ and ‘Right-only’), we applied an additional filter to remove false positives due to shorter motif sequences. Since the appropriate threshold for those half motifs has not been established, we calculated motif score-based thresholds by examining the distribution of binding sites with shared ChIP-seq experiments (Supplementary Fig. 1). RE1 half motifs with motif scores less than the thresholds were removed. Even after these stringent filtrations, we found relatively high numbers of RE1 half motifs compared to previous studies [6, 7, 10–12]. While it is possible that some of the RE1 half motifs we have identified may be false positives, a significant proportion of them are likely to be true positives, as they reflect the tissue specificity of RE1 motif profiles (Supplementary Fig. 2). Among 457 binding sites with full-length motifs, 350 (74%) sites showed canonical motifs with a regular length of gap (2 bp) (Fig. 2d). However, the ‘Convergent,’ ‘Divergent,’ and ‘Flipped’ forms displayed a greater incidence of altered gap lengths (Fig. 2d), implying that REST binding requires gap lengths that vary according to the specific conformation of the segments.

The distribution of RE1 motifs across exonic, intronic, and intergenic regions appeared to be consistent irrespective of the number of ChIP-seq experiments that shared peaks (Supplementary Fig. 3). This contrasts with a prior investigation [12], which reported a notable bias toward promoter regions of RE1 motifs shared across multiple tissues. This discrepancy may be attributed to differences in the respective annotation protocols employed. Specifically, our definition of ‘upstream’ incorporates a region spanning 1 kb from the transcription start site, while the definition of ‘promoter’ in the prior study may have encompassed a larger region, given the considerably greater proportion of ‘promoter’ sites (25–50%) compared to our ‘upstream’ sites (~ 3%).”

### Genome-wide RE1 motif profile

Through our analysis of 23 distinct human ChIP-seq experiments, we derived comprehensive genome-wide RE1 motif profiles (Fig. 3). As mentioned in the previous sections, canonical RE1 motifs (shown in black on the heatmap) were detected in REST ChIP-seq peaks that were universally observed throughout ChIP-seq experiments, while non-canonical RE1 motifs (shown in red-altered_gap, blue-convergent, green-divergent, purple-flipped, orange-L_only, and yellow-R_only on the heatmap) were identified in both universally observed REST ChIP-seq peaks and cell-line specific peaks. Interestingly, we identified a distinct cluster of universally observed REST ChIP-seq peaks that lacked RE1 motifs (Supplementary Fig. 2), which could potentially serve as promising candidate sites for novel REST binding motifs that differ from RE1 motifs.

It is notable that clear cluster patterns of ChIP-seq experiments by cell lines were observed (Fig. 3), with a few exceptions in brain cell lines (PFSK-1 and SK-N-SH) and one lymphoblast cell line of a leukemia patient (K562). Those exceptions might be resulted from protocol differences, since two different ChIP-seq protocols were used for each of the two experiments in these cell lines. Except for these cell lines, the other ChIP-seq experiments were well-clustered by their cell lines representing that REST binding has cell-line specificity for some binding sites. These distinct cluster patterns were primarily driven by a subset of ChIP-seq peaks that were shared by only a few experiments. Possible factors contributing to these cell-line specific bindings include variations in DNA methylation [18], chromatin status [19], and TF binding artifacts [17]. Notably, there were also many ChIP-seq peaks lacking RE1 motifs that were shared by only a few experiments (Supplementary Fig. 2). However, these peaks appeared to exhibit less cell-line specificity, as the experiments were not well-clustered based on their cell lines.

### Motif scores and TF binding

**Fig. 4:**
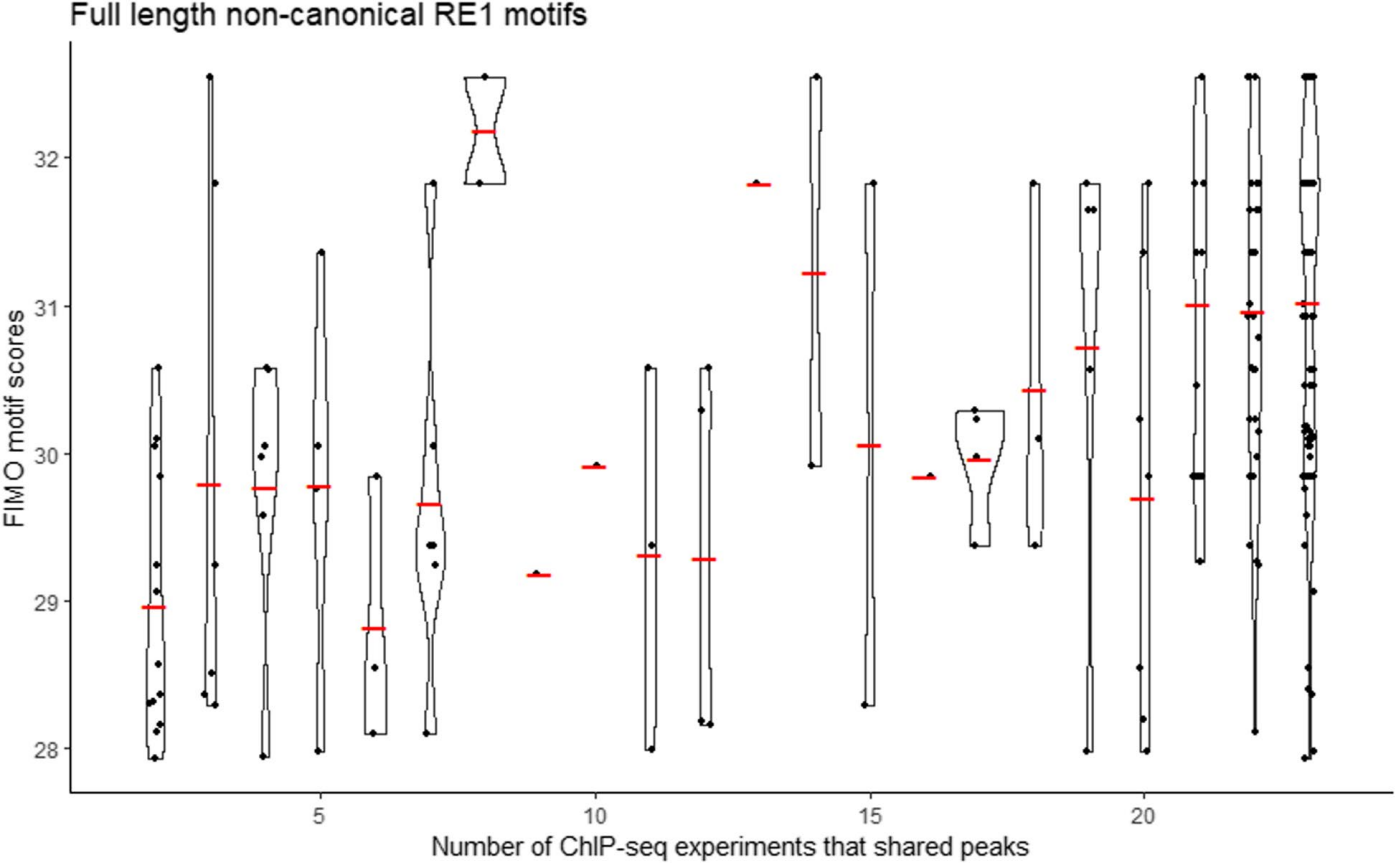
Motif scores and number of ChIP-seq experiments that shared peaks for full-length non-canonical RE1 motifs.  For the full-length forms (excluding ‘Left-only’ and ‘Right-only’ forms) of non-canonical RE1 motifs, the sum of FIMO motifs scores of two RE1 motif segments (left segment and right segment) by the number of shared ChIP-seq experiments are shown in violin and scatter plots. Red lines indicate mean values

Our analysis of all full-length RE1 motifs, excluding the ‘Left-only’ and ‘Right-only’ half motifs, revealed that RE1 motifs with higher motif scores are from ChIPseq peaks observed in many ChIP-seq experiments (Fig. 4). Furthermore, we observed that RE1 motifs from peaks called in more than 21 out of 23 ChIP-seq experiments had substantially higher motif scores compared to those with peaks in fewer experiments. These findings indicates that RE1 motifs similar to the consensus sequence have universal binding affinity, while variations in the motif sequence lead to cell-line specific TF bindings.

## Conclusion

We established a motif analysis method to analyze multiple sets of human REST ChIP-seq data from the ENCODE database to elucidate the characteristics of various RE1 binding motifs. Our findings demonstrated that canonical RE1 motifs exhibited widespread TF binding sites in most ChIP-seq experiments, whereas non-canonical RE1 motifs showed more varied binding sites observed both in multiple experiments and in specific cell-lines. We also discovered that each ChIP-seq experiment has a very distinct RE1 motif profile, even for the same cell-lines, and identified REST binding sites without RE1 motifs contributing to these differences. Furthermore, our analysis revealed a strong correlation between similarity scores of RE1 motifs to the consensus sequence and the number of ChIP-seq experiments that shared the peaks. Our comprehensive genome-wide profiling of RE1 motifs for REST binding sites will be a valuable resource to understand transcriptional or co-transcriptional regulation by REST.

To improve the quality of our motif analysis, we employed ENCODE blacklist [15] and HOT region [16] filtration and additionally filtered out ChIP-seq peaks found in only one experiment. We identified significantly more non-canonical RE1 half motifs than previously reported, which could be attributed to a lack of systematic motif search criteria for the half motifs in previous studies. The utilization of improved strategies to remove TF binding artifacts [17] might need to be applied to improve the overall robustness and accuracy of our findings.

Moreover, it is worth noting that recent studies have shed light on the potential for REST to bind to motifs other than RE1 motifs [12]. Our motif analysis showed a cluster of universal REST ChIP-seq peaks lacking RE1 motifs (shown in orange in Supplementary Fig. 2), which represent promising loci for the discovery of novel REST binding motifs that differ from RE1 motifs. Exploring these regions via motif enrichment analysis tools [20, 21] would be a valuable avenue for further investigation.

## Materials and methods

### ENCODE human REST ChIPseq datasets

Twenty three human REST ChIPseq peak call sets were downloaded in the narrowPeak bed format from the ENCODE database [13, 14] with the following identifiers: ENCFF039IZP, ENCFF087KDW, ENCFF096JDA, ENCFF200SDQ, ENCFF206NAT, ENCFF262MRD, ENCFF271FEM, ENCFF358XFJ, ENCFF526SUL, ENCFF539MIO, ENCFF622LOQ, ENCFF649VNE, ENCFF650LAZ, ENCFF668YET, ENCFF680JMZ, ENCFF707MDI, ENCFF717BYN, ENCFF738LQB, ENCFF814JWH, ENCFF866PJC, ENCFF884WVM, ENCFF975LZF, ENCFF994HAM (Supplementary Table 1). Overlapped peaks were merged by ‘multiinter’ and ‘merge’ functions from bedtools (version 2.27.1) [22].

### ENCODE blacklist and high occupancy target (HOT) region filtration

ENCODE blacklist region [15] and HOT region [16] information was downloaded from the ENCODE database [13, 14]. Peaks that mapped to HOT regions in any context with 5% significance combined metric (maphot_hs_selection_reg_cx_simP05_any.bed) or ENCODE blacklist regions (version v2) were filtered out using ‘subtract’ function with -A option from bedtools (version 2.27.1) [22]. Among 73,326 merged ChIPseq peaks, 4,351 peaks were filtered out, and 68,975 peaks remained after filtration.

### Identification of REST binding motifs (RE1 motifs)

REST binding motif information (ID: MA0138.2) was downloaded in the MEME format from the JASPAR database [23]. The whole motif was used for canonical RE1 motif search, and the half segments excluding the two bases in the middle were used for non-canonical motif search. Genomic regions of 68,975 merged ChIPseq peaks after HOT filtration were extracted from the GRCh38 human reference genome by ‘faidx’ function from SAMtools (version 1.3.1) [24] and were used as motif searching space input. The FIMO tool from MEME suite (version 5.3.3) [25] was used with default settings to search for both canonical and non-canonical forms of RE1 motifs.

For canonical motif search, the whole RE1 motif was used, and motif search results with their FIMO motif scores less than 84% of the maximum FIMO motif score were filtered out [26]. For non-canonical motif search, two half segments excluding two bases in the middle were searched separately. The left and right half segments of the RE1 motif were defined by the first 9 and the last 10 nucleotides, respectively (Fig. 2a). After filtering out motif search results with their FIMO motif scores less than 84% of the maximum FIMO motif score, motif search results for two half segments were merged based on their locations. When two motif search results with different segments were located adjacent to each other with gaps of 0 ~ 49 bases, they were merged as a pair. Merged motif search results were categorized into ‘regular’, ‘convergent’, ‘divergent’ or ‘flipped’ based on their orientations and locations. All the other remaining half segment results were categorized into ‘L_only’ or ‘R_only’. An additional motif score filter was applied to half segment RE1 motifs. ‘L_only’ motifs with FIMO motif scores less than 15 and ‘R_only’ motifs with FIMO motif scores less than 16 were filtered out.

### Genomic annotation

Genomic annotation was performed using ANNOVAR (version 20,170,601) [27].

### Heatmap of genome-wide RE1 motif profile

For each genomic regions of 68,975 merged ChIPseq peaks, the following categories were assigned for each of 23 ChIPseq experiments: (1) ‘NoPeak’ – no ChIPseq peak was found, (2) ‘Peak_NoRE1’ – a ChIPseq peak was found, but there was no RE1 motif, (3) ‘Peak_ncRE1’ – a ChIPseq peak was found with a non-canonical RE1 motif, and (4) ‘Peak_cRE1’ – ChIPseq peak was found with the canonical RE1 motif. A heatmap was plotted using ‘heatmap.3’ (https://github.com/obigriffith/biostar-tutorials/blob/master/Heatmaps/heatmap.3.R) with ‘fastcluster’ (version 1.2.3) [28] in R (version 3.5.1) [29].

### Supplementary Information

**Supplementary Material 1.**

**Supplementary Material 2.**

**Supplementary Material 3.**

**Supplementary Material 4.**

## Data Availability

The analysis result data will be provided after acceptance of the manuscript.

## References

[1] Chen ZF, Paquette AJ, Anderson DJ (1998). NRSF/REST is required in vivo for repression of multiple neuronal target genes during embryogenesis. Nat Genet.

[2] Lu T, Aron L, Zullo J, Pan Y, Kim H, Chen Y, Yang TH, Kim HM, Drake D, Liu XS (2014). REST and stress resistance in ageing and Alzheimer’s disease. Nature.

[3] Schoenherr CJ, Paquette AJ, Anderson DJ (1996). Identification of potential target genes for the neuron-restrictive silencer factor. Proc Natl Acad Sci U S A.

[4] Bruce AW, Donaldson IJ, Wood IC, Yerbury SA, Sadowski MI, Chapman M, Göttgens B, Buckley NJ (2004). Genome-wide analysis of repressor element 1 silencing transcription factor/neuron-restrictive silencing factor (REST/NRSF) target genes. Proc Natl Acad Sci U S A.

[5] Zheng D, Zhao K, Mehler MF (2009). Profiling RE1/REST-mediated histone modifications in the human genome. Genome Biol.

[6] Johnson R, Teh CH, Kunarso G, Wong KY, Srinivasan G, Cooper ML, Volta M, Chan SS, Lipovich L, Pollard SM (2008). REST regulates distinct transcriptional networks in embryonic and neural stem cells. PLoS Biol.

[7] Mouri K, Dewey HB, Castro R, Berenzy D, Kales S, Tewhey R (2023). Whole-genome functional characterization of RE1 silencers using a modified massively parallel reporter assay. Cell Genomics.

[8] Johnson R, Gamblin RJ, Ooi L, Bruce AW, Donaldson IJ, Westhead DR, Wood IC, Jackson RM, Buckley NJ (2006). Identification of the REST regulon reveals extensive transposable element-mediated binding site duplication. Nucleic Acids Res.

[9] Otto SJ, McCorkle SR, Hover J, Conaco C, Han JJ, Impey S, Yochum GS, Dunn JJ, Goodman RH, Mandel G (2007). A new binding motif for the transcriptional repressor REST uncovers large gene networks devoted to neuronal functions. J Neurosci.

[10] Johnson DS, Mortazavi A, Myers RM, Wold B (2007). Genome-wide mapping of in vivo protein-DNA interactions. Science.

[11] Rockowitz S, Lien W-H, Pedrosa E, Wei G, Lin M, Zhao K, Lachman HM, Fuchs E, Zheng D (2014). Comparison of REST cistromes across human cell types reveals common and context-specific functions. PLoS Comput Biol.

[12] McGann JC, Spinner MA, Garg SK, Mullendorff KA, Woltjer RL, Mandel G (2021). The genome-wide binding Profile for Human RE1 silencing transcription factor unveils a Unique Genetic Circuitry in Hippocampus. J Neurosci.

[13] An integrated encyclopedia (2012). Of DNA elements in the human genome. Nature.

[14] Luo Y, Hitz BC, Gabdank I, Hilton JA, Kagda MS, Lam B, Myers Z, Sud P, Jou J, Lin K (2020). New developments on the Encyclopedia of DNA elements (ENCODE) data portal. Nucleic Acids Res.

[15] Amemiya HM, Kundaje A, Boyle AP (2019). The ENCODE Blacklist: identification of problematic regions of the genome. Sci Rep.

[16] Wreczycka K, Franke V, Uyar B, Wurmus R, Bulut S, Tursun B, Akalin A (2019). HOT or not: examining the basis of high-occupancy target regions. Nucleic Acids Res.

[17] Carroll TS, Liang Z, Salama R, Stark R, de Santiago I (2014). Impact of artifact removal on ChIP quality metrics in ChIP-seq and ChIP-exo data. Front Genet.

[18] Yin Y, Morgunova E, Jolma A, Kaasinen E, Sahu B, Khund-Sayeed S, Das PK, Kivioja T, Dave K, Zhong F (2017). Impact of cytosine methylation on DNA binding specificities of human transcription factors. Science.

[19] Schmidt F, Gasparoni N, Gasparoni G, Gianmoena K, Cadenas C, Polansky JK, Ebert P, Nordström K, Barann M, Sinha A (2017). Combining transcription factor binding affinities with open-chromatin data for accurate gene expression prediction. Nucleic Acids Res.

[20] Mitra S, Biswas A, Narlikar L (2018). DIVERSITY in binding, regulation, and evolution revealed from high-throughput ChIP. PLoS Comput Biol.

[21] Frith MC, Saunders NFW, Kobe B, Bailey TL (2008). Discovering sequence motifs with arbitrary insertions and deletions. PLoS Comput Biol.

[22] Quinlan AR, Hall IM (2010). BEDTools: a flexible suite of utilities for comparing genomic features. Bioinformatics.

[23] Castro-Mondragon JA, Riudavets-Puig R, Rauluseviciute I, Berhanu Lemma R, Turchi L, Blanc-Mathieu R, Lucas J, Boddie P, Khan A (2021). Manosalva Pérez N : JASPAR 2022: the 9th release of the open-access database of transcription factor binding profiles. Nucleic Acids Res.

[24] Li H, Handsaker B, Wysoker A, Fennell T, Ruan J, Homer N, Marth G, Abecasis G, Durbin R, Subgroup GPDP (2009). The sequence Alignment/Map format and SAMtools. Bioinformatics.

[25] Grant CE, Bailey TL, Noble WS (2011). FIMO: scanning for occurrences of a given motif. Bioinformatics.

[26] Mortazavi A, Leeper Thompson EC, Garcia ST, Myers RM, Wold B (2006). Comparative genomics modeling of the NRSF/REST repressor network: from single conserved sites to genome-wide repertoire. Genome Res.

[27] Wang K, Li M, Hakonarson H (2010). ANNOVAR: functional annotation of genetic variants from high-throughput sequencing data. Nucleic Acids Res.

[28] Müllner D (2013). Fastcluster: fast hierarchical, agglomerative clustering routines for R and Python. J Stat Softw.

[29] Team RC (2018). R: a Language and Environment for Statistical Computing. In. Vienna.

