# Supplementary material for: Analysis of REST binding sites with canonical and non-canonical motifs in human cell lines
Source: BMC Med Genomics. 2024 Apr 17;17(Suppl 1):92. doi: 10.1186/s12920-024-01860-4 (PMC11025195; doi:10.1186/s12920-024-01860-4)
Supplement: Supplementary file 1 — Supplementary Material 1. [file 12920_2024_1860_MOESM1_ESM.docx]

**Supplementary Information**


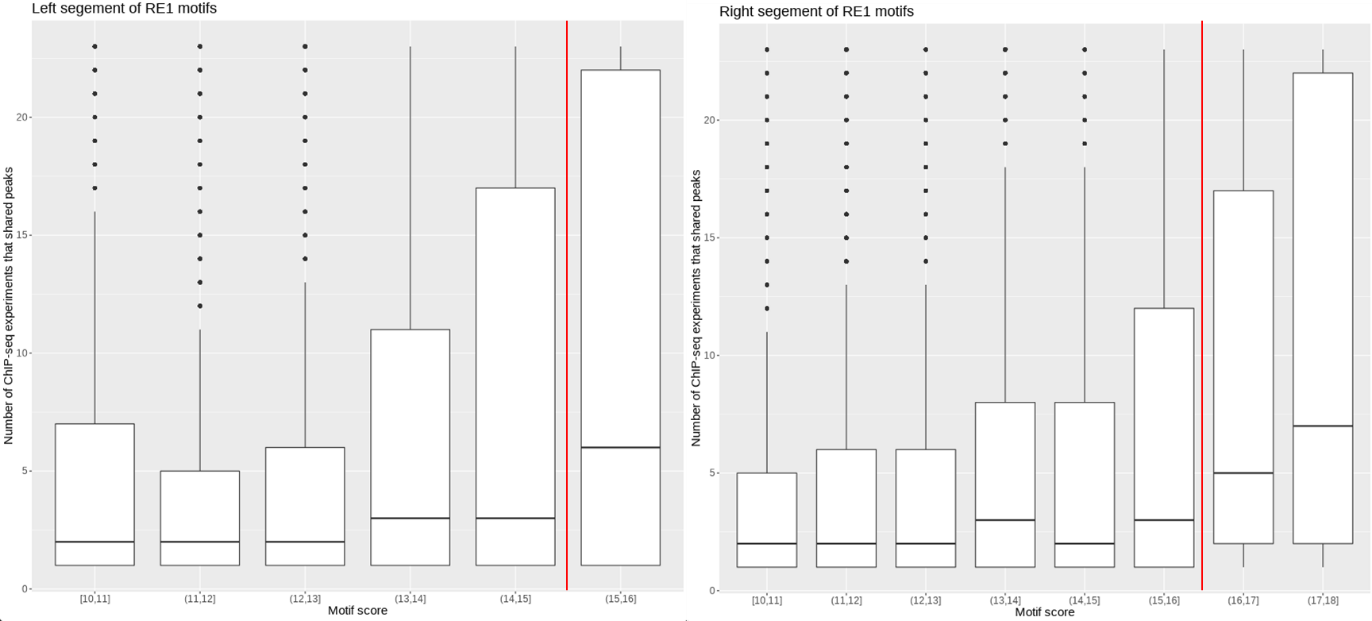


**Supplementary Figure 1 – Distribution of motif scores and numbers of REST ChIP-seq experiments with shared peaks with left or right only segments of RE1 motifs**

Box plots show the distribution of motif scores and numbers of ChIP-seq experiments that shared peaks with left or right only segments of RE1 motifs. Motifs with similar FIMO motif scores are merged with bin size of 1. Black horizontal lines represent medians, and red vertical lines indicate motif score thresholds, which were determined by examining the trend of median values and choosing a threshold to produce recurrent peaks in many ChIP-seq experiments.


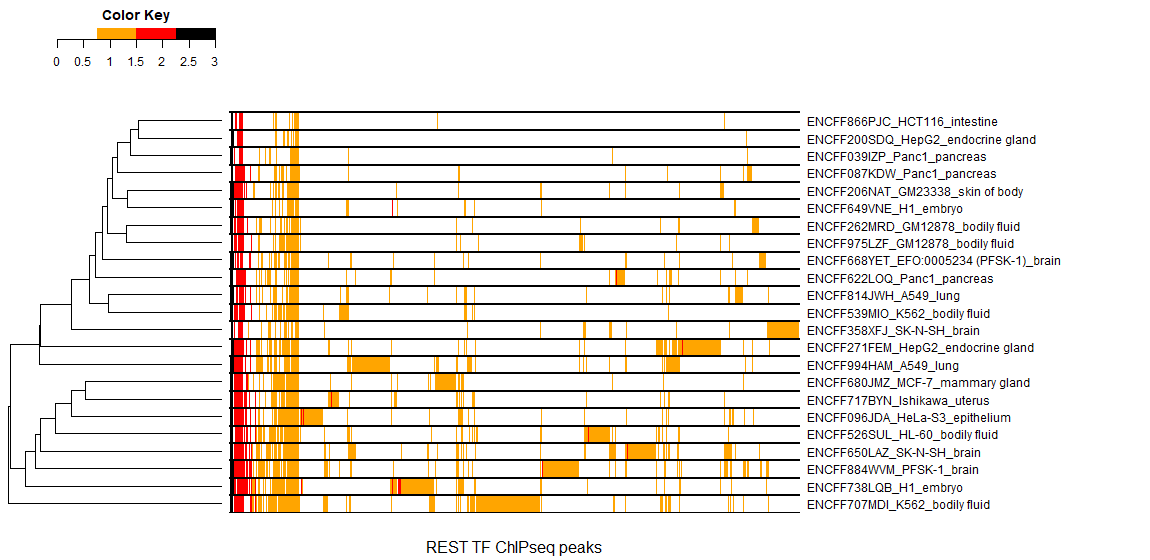

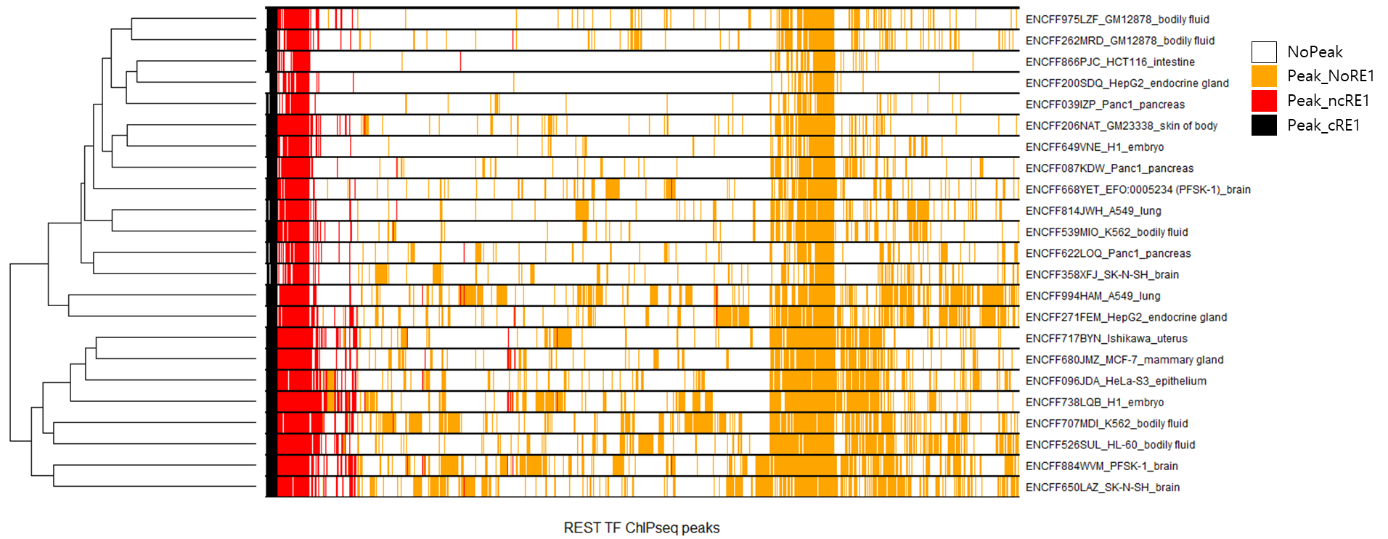


Peak_ncRE1:Altered_gap

Peak_ncRE1:Convergent

Peak_cRE1

NoPeak

Peak_ncRE1:L_only

Peak_ncRE1:R_only

Peak_ncRE1:Flipped

Peak_ncRE1:Divergent

**Supplementary Figure 2 – Genome-wide RE1 motif profile including peaks without RE1 motif**

The presented heatmap shows the genome-wide RE1 motif profiles of 68,975 REST TF ChIP-seq peaks from 23 ENCODE REST TF ChIP-seq experiments. The ChIP-seq experiments are identified through a three-segmented nomenclature, comprising the ENCODE identifier, cell-line name, and tissue name. Color key of heatmap - 1) White: ‘NoPeak’ – no ChIPseq peak was found in the relevant genomic region, 2) Orange: ‘‘Peak_NoRE1’ – ChIPseq peak was found in the relevant genomic region, but there was no RE1 motif, 3) Red: ‘Peak_ncRE1’ – ChIPseq peak was found in the relevant genomic region with non-canonical RE1 motif, but there was no canonical RE1 motif, and 4) Black: ‘Peak_cRE1’ – ChIPseq peak was found in the relevant genomic region with canonical RE1 motif.


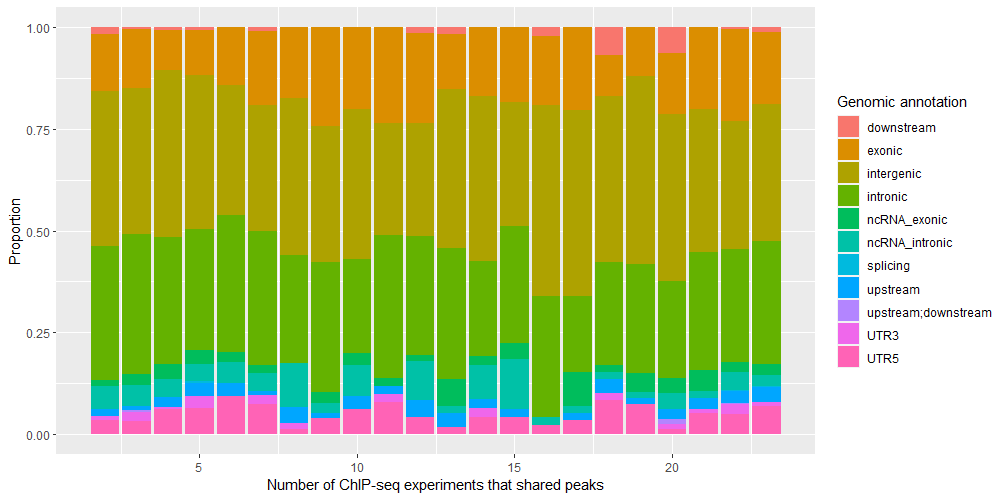


**Supplementary Figure 3 – Genomic annotation of RE1 motifs**

Proportions of each genomic annotations for each RE1 motif from ENCODE REST TF ChIP-seq peaks by their number of ChIP-seq experiments that shared peaks are shown as stacked bar plots.


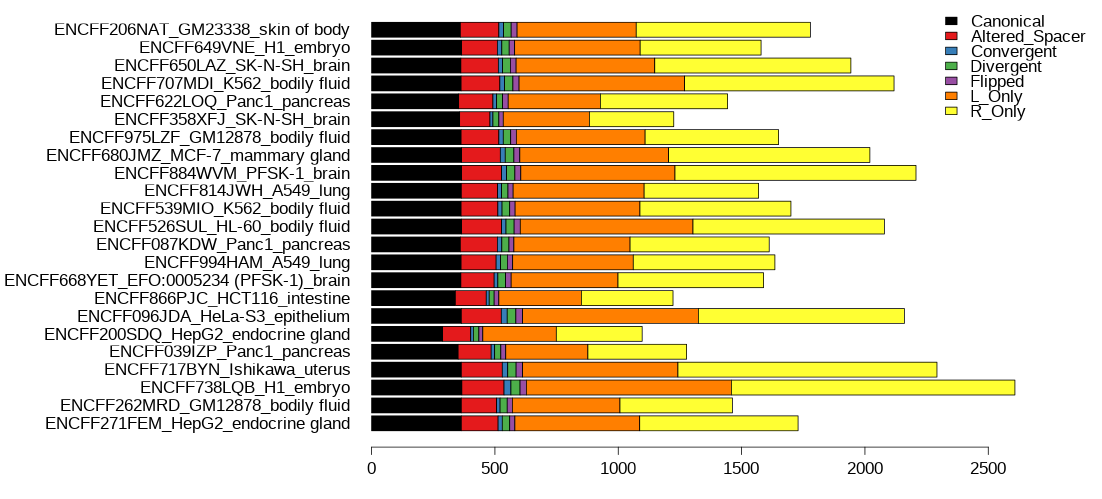


**Supplementary Figure 4 – Number of RE1 motif types from each ChIP-seq experiments**

Number of RE1 motif types from each ChIP-seq experiments are shown in stacked bar plots. X-axis indicates count of each motif types, and each experiment are displayed in each row.

**Supplementary Table 1 – Metadata table of ENCODE REST TF ChIPseq experiment (provided in a separate Excel file)**

Metadata information for 23 ENCODE REST TF ChIPseq experiments that were analyzed in our study is provided.

**Supplementary Table 2 – REST ChIPseq peaks with canonical RE1 motifs (provided in a separate Excel file)**

The table contains 350 REST ChIPseq peaks with canonical RE1 motifs. Column description: ‘Peak_location’ – Genomic location (GRCh38) of ChIPseq peaks; ‘count_chip’ – Number of ChIPseq experiments that shared peaks; ‘start’/’stop’ – Relative start/stop locations of RE1 motifs; ‘strand’ – Strand orientation of RE1 motifs; ‘score’ – FIMO motif search score; ‘matched_sequence’ – RE1 motif sequence of RE1 motifs; ENCODE narrowpeak filenames – If the relevant peak exists in the experiment, 1, otherwise, 0.

**Supplementary Table 3 – REST ChIPseq peaks with non-canonical RE1 motifs (provided in a separate Excel file)**

The table contains 2838 REST ChIPseq peaks with non-canonical RE1 motifs. Column description: ‘Peak_location’ – Genomic location (GRCh38) of ChIPseq peaks; ‘seg1_start’/’seg1_stop’ – Relative start/stop locations of the first segment of RE1 motifs; ‘seg1_strand’ – Strand orientation of the first segment of RE1 motifs; ‘seg1_score’ – FIMO motif search score of the first segment of RE1 motifs; ‘seg1_matched_sequence’ – RE1 motif sequence of the first segment of RE1 motifs; ‘seg2_start’/’seg2_stop’ – Relative start/stop locations of the second segment of RE1 motifs; ‘seg2_strand’ – Strand orientation of the second segment of RE1 motifs; ‘seg2_score’ – FIMO motif search score of the second segment of RE1 motifs; ‘seg2_matched_sequence’ – RE1 motif sequence of the second segment of RE1 motifs; ‘gap’ – Number of bases between two segments; ‘type’ – Type of non-canonical RE1 motifs; ‘count_chip’ – Number of ChIPseq experiments that shared peaks; ENCODE narrowpeak filenames – If the relevant peak exists in the experiment, 1, otherwise, 0.
